# The Impact of Situational Test Anxiety on Retest Effects in Cognitive Ability Testing: A Structural Equation Modeling Approach

**DOI:** 10.3390/jintelligence7040022

**Published:** 2019-09-23

**Authors:** David Jendryczko, Jana Scharfen, Heinz Holling

**Affiliations:** Institute of Psychology, Westfälische Wilhelms-Universität Münster, 48149 Münster, Germany; jana.scharfen@uni-muenster.de (J.S.); holling@uni-muenster.de (H.H.)

**Keywords:** retest effect, practice effect, cognitive abilities, intelligence, figural matrices, test anxiety, structural equation modeling

## Abstract

When a cognitive ability is assessed repeatedly, test scores and ability estimates are often observed to increase across test sessions. This phenomenon is known as the retest (or practice) effect. One explanation for retest effects is that situational test anxiety interferes with a testee’s performance during earlier test sessions, thereby creating systematic measurement bias on the test items (interference hypothesis). Yet, the influence of anxiety diminishes with test repetitions. This explanation is controversial, since the presence of measurement bias during earlier measurement occasions cannot always be confirmed. It is argued that people from the lower end of the ability spectrum become aware of their deficits in test situations and therefore report higher anxiety (deficit hypothesis). In 2014, a structural equation model was proposed that specifically allows the comparison of these two hypotheses with regard to explanatory power for the negative anxiety–ability correlation found in cross-sectional assessments. We extended this model for usage in longitudinal studies to investigate the impact of test anxiety on test performance and on retest effects. A latent neighbor-change growth curve was implemented into the model that enables an estimation of retest effects between all pairs of successive test sessions. Systematic restrictions on model parameters allow testing the hypothetical reduction in anxiety interference over the test sessions, which can be compared to retest effect sizes. In an empirical study with seven measurement occasions, we found that a substantial reduction in interference upon the second test session was associated with the largest retest effect in a figural matrices test, which served as a proxy measure for general intelligence. However, smaller retest effects occurred up to the fourth test administration, whereas evidence for anxiety-induced measurement bias was only produced for the first two test sessions. Anxiety and ability were not negatively correlated at any time when the interference effects were controlled for. Implications, limitations, and suggestions for future research are discussed.

## 1. Introduction

Taking the same or an alternate but equally difficult version of a cognitive ability test more than once has been observed to lead to an improvement in test performance—a phenomenon widely known as the retest effect [1] or practice effect [2]. The effect is psychometrically represented by a significantly increased (mean) test score or ability estimate upon a repeated measurement occasion. Retesting with cognitive ability tests can be crucial in clinical practice, personnel selection, and research scenarios. For example, in the evaluation of training procedures (e.g., for mathematical abilities), passive control groups often pass a simple retesting design to control for practice effects emerging from mere repetition rather than the training program.

The progression of retest effects over multiple test sessions and the causes for retest effects are not yet fully understood. Meta-analytic evidence for increasing test performance in various cognitive ability tests due to retesting has been reported by several authors [2,3,4,5,6]. The size of the effect is moderated by several variables such as equivalence of test forms, test-retest interval, participant age, as well as cognitive ability operation and content. Scharfen et al. [6] observed that scores in a wide range of cognitive ability tests increased up to the third test administration where they seemed to reach a plateau. Cognitive ability tests included in this analysis were general memory, processing speed, divergent thinking, and reasoning. A non-linear progression of retest effects has also been found by a meta-analysis focusing on score gains in working memory tests [5]. Finally, retest effects in figural matrices tests seem to follow this pattern as well [6]. This is especially noteworthy because figural matrices test scores are highly correlated with psychometric *g*, the construct of general intelligence [7,8]. However, these meta-analyses only included a low number of studies that administered more than three tests and the authors recommended that researchers investigate retest effects across several test administrations in the future.

The studies in which cognitive ability tests were administered more than three times [9,10,11,12,13,14,15,16,17,18,19,20] are heterogeneous with regards to the population of interest, the cognitive abilities investigated, the applied measures, the use of parallel test forms, the length of the test-retest intervals, considered covariates of retest effect-size, and the main research question. For instance, Kaminski et al. [15] and Fredrickson et al. [14] were interested in the stability of scores of specific neuropsychological test batteries; Falleti et al. [12] and Bartels et al. [10] explored the influence of the test–retest interval on retest effect size. Other important covariates of retest effect size were considered by Ferrer et al., Rogers et al., Wilson et al., and Wilson et al. [13,17,19,20]. The authors either studied group differences of retest effects in neuropsychological testing between healthy participants and participants with brain injuries [17,19] or separated incremental effects of age and aging from actual practice effects [13,20].

Despite the diversity of these studies, they all share some attributes that have important implications for future studies on retest effects. None of these studies found a significant increase in test scores after the seventh test administration. This suggests using seven test sessions as a general guiding value for longitudinal studies on the progression of retest effects. No study investigated retest effects specifically in the measurement of psychometric *g*. Most of the studies focused on basic neuro-cognitive assessments such as reaction time, matching, and monitoring. Although the investigation of these abilities is a worthwhile task, *g* is the central variable in cognitive ability assessment settings for personnel selection and achievement prediction (although other valid predictors exist [21]). A comprehensive assessment of intelligence requires the use of complete test batteries that include various ability measures, which are impractical for longitudinal studies, as they impose a high burden on participants when test–retest intervals are not particularly long. However, valid proxy measures of *g* reflect a compromise in this context. Puddey et al. [16] investigated retest effects within the undergraduate medicine and health science admission test (UMAT). The first subscale of this test measures fluid reasoning, which provides the highest correlation with *g* among the more specific ability factors and is hence often used as a proxy IQ measure [7,8]. This study was, however, practically-oriented. The covariates of retest effect sizes that were considered included, for example, testees’ nationality and first language, because these have important implications for fairness in student selection. However, they do not provide a theory-driven explanation for the emersion and development of retest effects.

Accordingly, studies on retest effects are noticeably lacking that simultaneously incorporate: (1) the assessment of general intelligence or at least the use of valid proxy measures, (2) the description of retest effect development beyond the third test session, and (3) an explanation of this development within a psychological theory.

Statistical modeling of such a research scheme is not straightforward. A change model must be employed that quantifies the changes in ability estimates upon retesting to comply with points (1) and (2). The model must include covariates of the ability to account for point (3). Exact model specifications largely depend on the proposed relationship of the retest effects and the covariates of the ability. Structural equation modeling (SEM) is potentially flexible enough to accommodate these conditions.

The purpose of this study was two-fold. Firstly, we derived a statistical model for the investigation of retest effects that considers situational test anxiety as their explanation. Test anxiety was the covariate of choice because a literature review revealed the potential for this predictor on the theoretical level and an opportunity for a solid statistical modeling of its impact on retest effects in the frameworks of SEM. Secondly, a longitudinal empirical study with seven proxy measurements of *g* incorporating this methodological approach was conducted. 

The rest of this article is thus structured as follows: First, a review of explanations for retest effects is provided. The potential and particularities of test anxiety are stressed. Second, the literature on the anxiety test performance relationship is illustrated in more detail and a theory for the impact of situational test anxiety on retest effects is proposed. Lastly, the statistical model representative of this theory is derived and the study is presented.

### 1.1. Explanations for Retest Effects

Lievens et al. [22] introduced a theoretical framework differentiating between three groups of causes leading to retest effects. More recent theoretical reviews have built upon this framework from different perspectives [23,24]; yet, they acknowledged the following three categories to be the most important: (1) Retest effects can reflect a gain in the measured latent ability. In contrast to the other two explanations, this hypothesis does not assume other states or traits apart from the cognitive ability itself to be involved in the observed increase of test scores. Frequent amplified usage of the cognitive ability due to retesting causes a training effect, so that an increased test score manifests as the result of an improvement in ability. (2) Test-specific strategies and skills (in contrast to the actual latent ability measured by the test) are assumed to be fostered by retaking a test, leading to higher scores. For example, across multiple test sessions, a testee could learn how to conclude the right answer to a multiple-choice test by excluding the wrong answers instead of engaging in the task itself. The capability to exploit such test-specific strategies to receive a higher score is often referred to as test-wiseness [25]. (3) The influence of construct-irrelevant factors, such as test anxiety, unfamiliarity, and rule incomprehension, which initially prohibit testees from performing at their true level of ability, can be reduced due to retesting.

These three categories can be differentiated in terms of their implications for the consequences of retesting on the construct validity of a cognitive ability test. The first cause, increased latent ability leading to higher scores, would have no consequences on the construct validity of the test as it still measures the underlying ability without deterrence due to retesting. In the second case, when test-specific strategies and skills are fostered, the measurement of the latent ability is deterred due to retesting, meaning that taking a test repeatedly leads to lower construct validity. In contrast, as construct-irrelevant factors are reduced, construct validity is enhanced due to retesting. Yet, conclusions on the weights of these potential causes cannot be drawn as empirical evidence is mixed with regards to whether construct validity is influenced by retesting [9,26,27,28,29].

Randall and Villado and van Iddekinge and Arnold [23,24] expanded Lievens et al.’s [22] framework by explicitly reviewing specific variables that possibly explain retest effects. The authors concluded that some factors seem to moderate the size of the retest effects (e.g., demographics, test-wiseness, and equivalence of test forms), whereas more research is needed for most of the construct-irrelevant factors. This is especially the case for motivational states and emotions that influence (re)test performance. This conclusion was also drawn by Scharfen et al. [6]. The authors introduced theoretical deliberations on how motivational and emotional states relating to test achievements might change over multiple test sessions. For example, it was argued that test anxiety strongly inhibits test performance at the first test administration but gradually stabilizes with successive test repetitions. In this context, Van Iddekinge and Arnold [24] specifically stressed that anxiety scores were found to decrease upon retesting [30,31] and that a long line of research has been dedicated to the relationship between test anxiety and test performance (see below); however, research on the effect of test anxiety on ability score changes is still missing. Matton et al. [32] specified a longitudinal SEM approach in which the impact of construct-irrelevant factors on ability estimate changes across time can be tested. They demonstrated how a general residual factor representative of such construct-irrelevant variables can be added to the model equations and estimated (see also [33]). Test anxiety was mentioned as a central theoretical component cumulated in that factor but not assessed directly. Similarly, Reeve et al. [34] emphasized the importance of an exact statistical specification of the influence of construct-irrelevant factors on ability test performance. They reported an example of the impact of test anxiety and test familiarity on ability scores in the framework of classical test theory and elucidated the implications of these specifications with a Monte Carlo simulation. Again, test anxiety was not assessed directly.

The specific construct of test anxiety has, however, been studied since 1952 [35] and is now well established, measurable, and incorporated into various theories on cognitive ability test performance. These theories have been recently translated into concrete statistical formulas in the framework of SEM [36] and can be extended to longitudinal research on test performance improvement. It follows that the request for a more precise insight into the incremental contribution of test anxiety to the emersion and progression of retest effects can now be pursued.

### 1.2. Definition of Test Anxiety

Broadly, test anxiety refers to a facet of general anxiety that is specific to evaluative situations [37,38,39]. As with general anxiety, an important distinction is made between trait test anxiety and state test anxiety. The former reflects a quite stable dispositional proneness to anxious thoughts and feelings when being tested [40,41]. The latter is more fluctuating and dependent on the attributes of a specific test situation [40,42]. For this reason, state test anxiety is also often referred to as situational test anxiety (STA) [43,44]. More recently, researchers have started to emphasize the incremental effects of trait and state anxiety on test performance [38,45,46,47,48], but most research so far has focused on the link between STA and test scores [39]. STA is often operationalized in the framework of current achievement motivation (CAM) [49,50,51]. For example, the questionnaire for current achievement motivation [52] includes the sub-facet fear of failure (FOF), which reflects worrisome cognitions and beliefs toward the test outcome in a specific evaluative situation and can be considered a measure of STA.

### 1.3. Test Anxiety and Test Performance: Interference and Deficit Hypotheses

Meta-analytic evidence suggests that test anxiety and test performance are negatively correlated [53] and subsequent empirical work further supports this finding [36,38,44,46,54,55,56]. However, the nature of this relationship is still debated. Two contrary approaches compete as potential explanations: the interference hypothesis and the deficit hypothesis.

The interference hypothesis claims that anxiety reduces test performance. High levels of anxiety prevent testees from performing at their true level of ability because cognitive resources are depleted for the processing of and/or emotional coping with worrisome thoughts about the test outcome [57,58,59]. Hence, the interference hypothesis suggests that measurement bias evolves due to the influence of anxiety [38,44].

Conversely, the deficit hypothesis postulates no causal relationship between test anxiety and performance. The negative correlation between anxiety and test performance emerges merely due to an activation of test anxiety by the confrontation with an ability test. If a person generally achieves high test scores, they might report less anxiety toward testing situations. In contrast, those testees that perform worse in testing scenarios become aware of their deficits when confronted with a test and report higher test anxiety [38,39,56,60,61,62,63,64,65,66,67,68]. More refined versions of this hypothesis explicitly trace the correlation between test performance and test anxiety back to the attenuating third variable, test competence [54,69,70,71]. Most importantly, in contrast to the interference hypothesis, the deficit hypothesis assumes no cognitive ability measurement bias due to anxiety.

**Figure 1:**
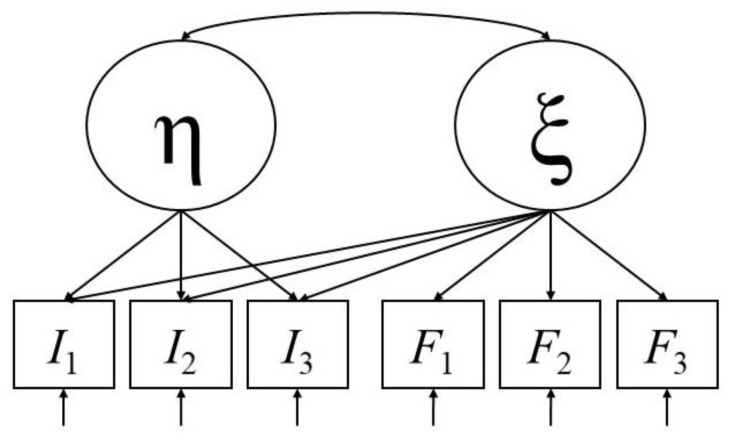
Anxiety test (AT) model as proposed by Halpin et al. [36]. A latent cognitive ability variable (η) is measured by its respective manifest test items (three in this example: *I*_1_, *I*_2_, and *I*_3_). A latent anxiety variable (ξ) is measured by its respective manifest questionnaire items (again, three in this example: *F*_1_, *F*_2_, and *F*_3_). The arrows approaching the manifest variables from below represent their respective error terms. Regressions of the ability test items on latent anxiety are also modeled and reflect interference effects. The correlation between ability and anxiety is modeled and represents a potential deficit. Simultaneous modeling of interferences and deficits creates statistical rotational indeterminacy, rendering the model under-identified. However, strategic equality constraints among the factor loading parameters (for example: all interference effects are restricted to the same value) solve this problem.

The superiority of either hypothesis in an empirical context has been evaluated by investigating the presence of anxiety-induced measurement bias and results have been mixed [36,38,44,46,56,65]. For that purpose, Halpin et al. [36] introduced a methodological framework to simultaneously assess the interference and the deficit hypothesis on the basis of structural equation modeling. In this anxiety test model (AT model, Figure 1), a latent cognitive ability variable is measured by the ability test items, and a latent trait or state test anxiety variable is assessed, for example, by proper questionnaire items. Latent anxiety also negatively influences the ability test items in accordance with the interference hypothesis because high anxiety should causally reduce the probability of solving these items correctly. A negative regression coefficient of a test item on latent anxiety thereby reflects systematic measurement bias and is described as an interference effect. The correlation between latent ability and latent anxiety is modeled in accordance with the deficit hypothesis. If interference effects are found and this correlation is significantly lower than zero, then both interference and actual deficits should be assumed to explain the test outcome. If this correlation decreases to zero, when interference is controlled for, only evidence for the interference hypothesis can be drawn from the model. Finally, if no interference effect is found but a negative correlation between the latent variables still emerges, the deficit hypothesis can be seen as the next best explanation for this correlation. As Halpin et al. [36] pointed out, this simultaneous modeling of double loadings for the ability test items and the correlation of the latent variables creates statistical rotational indeterminacy and leaves the model unidentified. However, they also demonstrated that strategically imposed equality constraints among factor loading parameters solve this problem.

### 1.4. A Psychological Theory for the Impact of Situational Test Anxiety on Retest Effects

To the best of our knowledge, the interference and the deficit hypotheses have been evaluated as explanations for test performance before, but not for retest effects. In accordance with the deliberations introduced by Randall and Villado [23] and Scharfen et al. [6], measurement bias due to anxiety, as postulated in the interference hypothesis, might be high in earlier test sessions when participants do not know what to expect from the test. However, it might play less of a role in later test sessions when participants have grown accustomed to the test situation and are no longer alarmed by it. This formulation does not yet explicitly postulate whether the impact of state (i.e., situational) or trait test anxiety on test performance diminishes with ongoing test repetitions. However, work has been published that relates a decrease in anxiety to a habituation process occurring due to frequent exposure to test situations [6,72,73]. Habituation reflects a flattening effect on a temporally and contextually fluctuant (emotional) reaction. This kind of reaction dynamic is more accurately represented by the concept of situational test anxiety as opposed to rather stable trait test anxiety. We therefore focused on STA in the current work but elaborate upon the significance of trait test anxiety in the discussion.

The decrease in STA interference over time explains the increase in test performance over time. The larger the reduction of interference (i.e., measurement bias) between two successive test sessions, the larger the expected retest effect. This also implies an increase in cognitive ability measurement reliability and validity over time. Oostdam and Meijer [74] investigated the intra-individual change in anxiety-induced interference within a single assessment session under a comparable theoretical umbrella, but they did not specifically relate changes in interference to retest effects.

To evaluate this theory, we suggest a method of extending the AT model so that it can be employed to longitudinal data. In the following, we describe this extension and then demonstrate its usage in a longitudinal study.

## 2. A Statistical Model for the Impact of Situational Test Anxiety on Retest Effects

**Figure 2:**
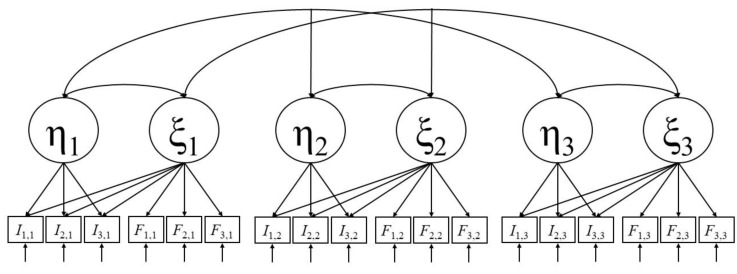
Extension of the AT model [36] to longitudinal data (LAT model). An AT model (Figure 1) is constructed for every test session *i* (three in this example but it can be extended to any number of test sessions). States of latent cognitive ability (η*_i_*) are modeled to correlate between test sessions. The same is applied to latent state anxiety (ξ*_i_*).

In a first step, repeated measurements of cognitive ability and situational test anxiety have to be introduced to the original AT model. For that purpose, an AT model is constructed for every test administration. Combining these models requires theoretical deliberations on the specification of latent variable correlations. At every test session, latent ability and latent anxiety are modeled to correlate with each other to account for deficit effects. Latent ability states are modeled to correlate across test administrations to account for construct stability. The same is applied to latent anxiety states. Figure 2 depicts an example of this extension of the AT model. We refer to it as the longitudinal AT (LAT) model.

Although this model allows for an investigation into interference development, it does not consider retest effects. To compensate for this, the modeled relationships of the latent ability variables can be further extended to a latent growth curve in terms of the neighbor-change model [75,76,77,78] (for an illustration see [79]). This procedure introduces latent ability difference variables between each pair of successive test sessions to the model. These difference variables represent individual ability estimate changes between two successive test administrations. These changes are computed by residual-free regressions of later ability states to latent ability at T1 and all previous difference variables. All regression weights are constrained to 1. Let *η_k_* represent the latent ability state at T*k* (*k* ≠ 1) and let *δ_i_*_+1,*i*_ represent the latent difference variable between T*i* and T*i* + 1, then for all *η_k_*:

(1) $$\eta_{k}=\;\eta_{1}+\;\overset{k-1}{\underset{i=1}{\sum }}\delta_{i+1,i}$$

holds. From this equation, we can deduce:

(2) $$\delta_{k,k-1}=\;\eta_{k}-\eta_{k-1}.$$

For example, in a case with three test sessions, latent ability at T2 is defined as:

(3) $$\eta_{2}=\;\eta_{1}+\delta_{2,1}$$

and latent ability at T3 is defined as:

(4) $$\eta_{3}=\;\eta_{1}+\delta_{2,1}+\delta_{3,2}.$$

Solving Equation (3) for *δ*_2,1_ gives:

(5) $$\delta_{2,1}=\;\eta_{2}-\eta_{1}.$$

Inserting the right-hand side of Equation (5) into Equation (4) and solving for *δ*_3,2_ gives:

(6) $$\delta_{3,2}=\;\eta_{3}-\eta_{2}.$$

These equations display how this procedure computes the latent ability changes between two successive test administrations for each individual testee. Accordingly, the means of these latent difference variables reflect the mean retest effects between two successive test sessions. When the model is identified by constraining the variances of the latent variables to 1, the latent difference variables are on a standardized scale and the mean retest effects can be interpreted as effect sizes in terms of Cohen’s *d* [80]. Note, this computation of retest effects requires strong longitudinal measurement invariance of the cognitive ability test because the means of the latent difference variables cannot be estimated and do not have a meaningful interpretation unless intercepts/thresholds of the respective manifest variables are kept constant across test sessions. For further explanations on measurement invariance see Section 3). Correlations among latent abilities across test sessions are replaced by this growth curve in the LAT model. The latent difference variables are modeled to correlate with latent ability at T1 and with each other. These correlations cannot be interpreted as stabilities of latent states. This model thereby reflects a special case of a latent growth curve model with a random intercept (*η*_1_) and correlated slope variables (the latent difference variables).

**Figure 3:**
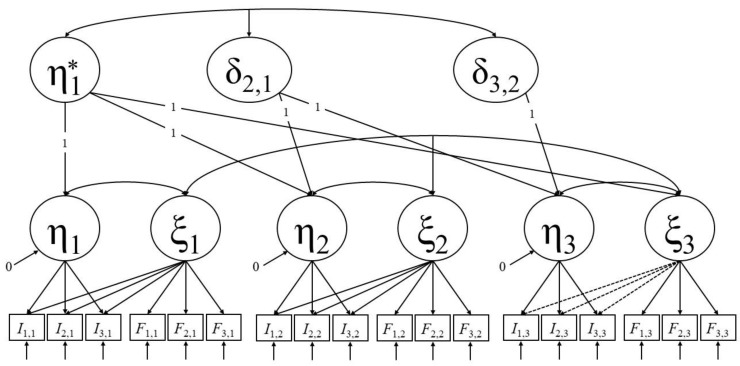
Example of the full interference model with three test sessions. ξ*_i_* (with *i* = 1, 2, 3) depicts situational test anxiety at measurement occasion *i*. We included a latent variable η^*^_1_ for a more comprehensible visualization of the model. η_1_ regresses on η^*^_1_ with a regression weight of 1 and a residual-variance of 0. Hence, η^*^_1_ = η_1_. Note that an inclusion of η^*^_1_ is not necessary for model estimation as η_1_ can be used for the respective equations instead. By identifying the model by setting the variances of latent variables to 1, the latent differences variables δ*_k,k−1_* (with *k* = 2, 3) are on a standardized scale and their means can be interpreted as retest effect sizes in terms of Cohen’s *d* [80]. Interference effects in the third test session are depicted by dashed lines to illustrate the first step of the interference reduction approach. A model in which these coefficients are restricted to zero can be compared with the full interference model via a likelihood ratio test to test the null hypothesis that interferences disappear in the third test session.

The model contains both retest and interference effects. A change in the amount and overall size of interference effects between two successive test sessions can be set in relation to the retest effect between these two test administrations. Since interference effects are estimated at every test session in this model, we call it the full interference model. However, the model can be restricted by setting the interference effects of the last test session to zero, thereby creating a more parsimonious nested model. By comparing both models with a likelihood ratio test, the null hypothesis that no interference occurs at the last test session is tested. Next, the interference effects of the second to last test session can be additionally restricted to zero. This model can then be compared with the model in which only interferences in the last test session are assumed to be absent. This procedure can be successively continued until all interference effects are restricted to zero. If anxiety-induced measurement bias only exists up to a certain test session, this session will be revealed by this procedure. The values of the Δχ^2^ test statistic of the likelihood ratio tests can be compared in size to reveal information about the magnitude of interference in a given test session. This occurs because, with every step, the same amount of additional interference effects is restricted to zero given that the number of test items is kept constant across test administrations, keeping the degrees of freedom constant across all likelihood ratio tests. The results of these successive tests can be interpreted in the light of the observed retest effects, as a comparably large retest effect between T*k* − 1 and T*k* should align with a comparably large Δχ^2^ test statistic when restricting interference effects at T*k* to zero. In the following, we refer to this framework as the interference reduction approach. Figure 3 depicts an example of the full interference model with three test administrations and visualizes the first step of the interference reduction approach.

## 3. An Empirical Study

We investigated the interference reduction approach in a longitudinal SEM framework. First, an intelligence test was administered seven times to explore the longitudinal development of retest effects. For economic reasons, we did not want participants to undertake a complete intelligence test battery that often. Hence, the cognitive ability test of choice was a figural matrices test that is particularly useful for longitudinal studies because it contains parallel forms (see Section 3.1 and Supplementary Materials (Appendix A)). Figural matrices tests measure fluid reasoning, which has the highest *g* saturation of all of the more specific cognitive ability factors. They are arguably the best IQ proxy measure when only a single measure can, or should, be used [7,8]. Second, situational test anxiety was assessed at every test session to investigate potential interference on test scores and their relation to retest effect size. This study thereby reflects one of the first attempts to simultaneously explore both of the following: retest effects within the measurement of *g* beyond the third test session, and the construct-irrelevant factor of test anxiety as a potential explanation for retest effects.

### 3.1. Method

#### 3.1.1. Sample

Participants were mainly recruited among students of the University of Münster, Germany via flyers and invitations on social media channels in 2017. They had the option to select between various combinations of monetary remuneration and course credit, with a maximum monetary remuneration of €50. A total of 326 examinees originally participated in the study. Out of these, 297 completed every test session. This reflects a dropout rate of 8.9%. Another 21 further examinees had to be excluded because they required more than three hours for one of the tests, which questions the validity of their data. From the remaining 276, further participants were excluded to account for ceiling effects in longitudinal test score development. Ceiling effects were defined as yielding a perfect test score in four or more test sessions or yielding three perfect test scores in a row. The data of the remaining 225 examinees were used for the analysis (see Supplementary Materials (Appendix B) for analysis of the complete sample). In this sample, 24.9% reported being male and 74.7% being female (one missing value). The mean age was 23.49 (SD = 4.84). Of the participants, 45.33% studied psychology, 8.44% studied economics, 2.67% studied communication science, and 10.67% were not students.

#### 3.1.2. Measures

##### Figural Matrices Test

The figural matrices test was an updated version of MatrixDeveloper, which was described in detail by Freund et al. [81]. Per test session, 13 items had to be solved. With every item, a 4 × 4 matrix was presented to the participants. The matrix was filled with symbols following certain rules that were applied row-wise. The cell on the bottom-right of the matrix contained a question mark. Participants had to identify the correct cell to replace that question mark according to the symbol rules out of the 16 options that were displayed below the matrix. One of the options was “No option is correct”. The time limit was set to three minutes for each matrix item. Feedback on whether the given answer was correct was provided immediately. For any given item, participants scored one point when the correct answer was selected and zero points for the wrong answer.

MatrixDeveloper is software that generates a test on the basis of rule-based automatic item generation [82,83]. The rules that dictate the symbol patterns are considered the radicals of the test and determine the psychometric properties of the items. The test creator can decide which of the six rules shall be active in any given item. The color (either black or white), shape, and amount of symbols are randomly chosen to follow an activated rule. They are considered the incidentals of the test that have no effect on the psychometric item properties. This item generation procedure theoretically allows for the creation of a nearly infinite number of parallel test forms.

The rules that determine symbol patterns are explained to the testee before the test administration. In other words, this matrices test does not require participants to discover the rules themselves but to recognize the already known rules in symbol patterns and to logically conclude the correct answer. For a detailed explanation of these rules, refer to Freund et al. [81].

Since presenting the exact same test several times confounds underlying retest effects of interest with mere memory effects of already seen test items, we aimed to create seven different but equally difficult tests. Thus, items were selected via a matching system: easiness (probability of solving an item correctly given average cognitive ability) parameters of a pool of items were available from a calibration study. Each test session’s items were selected so that their easiness parameters covered a wide range of ability. We ensured each item for a specific test session had a matched item in every other test session that had a similar difficulty. Since at every test session items were presented in order of increasing difficulty, the match of an item from one test session was always presented in the same position (from 1 to 13) in any other test session. The mean easiness for the 13 items belonging to a respective test session ranged from *Min*(*M*) = 0.551 to *Max*(*M*) = 0.558 (*M*(*M*) = 0.554) and the standard deviation for item easiness in a test ranged from *Min*(*SD*) = 0.241 to *Max*(*SD*) = 0.271. For a more detailed description of the test item easiness parameters see Supplementary Materials (Appendix A).

##### Situational Test Anxiety

Situational test anxiety was assessed in the framework of current achievement motivation (CAM) [49,51]. STA was measured with the subscale fear of failure (FOF) of the German version of the questionnaire for CAM (“Fragebogen zur Erfassung aktueller Motivation (FAM)” [52]) by five statements. Participants rated these statements on a 7-point-Likert scale, ranging from “I strongly disagree” to “I fully agree”. One example statement is: “When I think of these tasks, I feel a little disconcerted”.

#### 3.1.3. Procedure

Data were collected via an online survey. The time interval between subsequent test sessions was three to four days. Three days after completing a test session, participants received an email with the link to the next test, which was required to be undertaken within the next 48 h. Participants’ email-addresses were solely used for distributing the links to the later test administrations. They were deleted after the end of the field time of the study.

In every test session, participants were greeted and informed about the study purpose, anonymity of participation, the alternatives for reimbursement, and the requirement for study completion to receive any form of remuneration. They were further informed that by proceeding, they consented to participation and use of their anonymized data for analysis. They first had to provide their email address and generate (in the first test session) or enter (at the subsequent test sessions) their participant code. Next, the figural matrices test and the item rules were explained. Afterward, the FAM was administered. Then, participants again received a short overview of the matrices test rules. This was followed by the figural matrices test. Afterward, participants had the opportunity to leave comments.

During the course of the seven test sessions, the procedure varied in some cases. In the first session, every matrices test rule was explained in detail and every explanation was followed by a training exercise to ensure complete understanding of the respective rule. Participants that failed to answer any training item correctly within the first three attempts were prohibited from continuing. In the remaining test sessions, the rules were only briefly repeated, and no practice items were presented. At the end of the first session, demographic information was collected, and at the end of the seventh test session, participants could choose their combination of monetary and course credit reimbursement. Three weeks after the study field time closure, compensation was distributed to all participants.

#### 3.1.4. Analytic Strategy

Data were analyzed using SEM with the R [84] package lavaan version 0.6-3 [85]. Generally, in all models containing the binary answers to figural matrices items (0 = wrong answer, 1 = correct answer), parameters were estimated with the DWLS discrepancy function [86,87], whereas robust standard errors were obtained via the WLSMV method. Parameters and standard errors of models containing only the FOF-scale items were estimated using the robust maximum likelihood (MLR) method. For all models, fit was evaluated via the χ^2^ overall model fit test statistic, χ^2^-to-*df* ratio, RMSEA, CFI, and TLI. The cut-off criteria for all these fit indices were taken from West et al. [88]. For the nested models, we used likelihood ratio tests according to the Satorra method [89] for DWLS-estimated models, and the Satorra–Bentler method [90] for MLR-estimated models. Additionally, we compared CFI values. A decrease in CFI larger than 0.01 after imposing restrictions on model parameters is considered a substantial decline in model fit [91].

**Figure 4:**
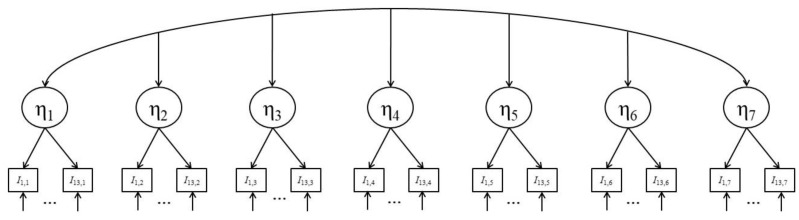
Cognitive ability-confirmatory factor analysis (CFA). η_1_–η_7_ represent the latent ability variables measured by the figural matrices test items in every test session. *I*_1,1_ represents the first item in the first test session, *I*_13,7_ represents the 13th item of the seventh test session, etc. (items 2–12 of any test session are not shown but are represented by the respective three dots). Factor loadings can vary without any restrictions in a configural invariant model, but the loading of any item is restricted to the same respective value across test administrations when a more restrictive from of invariance is implemented. The threshold of any test item (not shown) is also restricted to the same respective value across test administrations when strong invariance is imposed. The arrows approaching the manifest variables from below represent their respective error-terms. The model was identified by setting the factor loading of the first item at every test session to 1.

**Figure 5:**
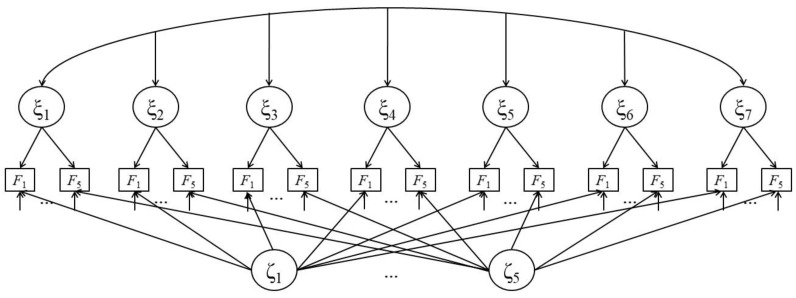
**Situational test anxiety-confirmatory factor analysis** (STA-CFA). ξ_1_–ξ_7_ represent the latent STA variables measured by the fear-of-failure (FOF) items of the “Fragebogen zur Erfassung aktueller Motivation” (FAM) at every test session. *F*_1_ represents the first item of the questionnaire and *F*_5_ represents the fifth item. Items 2 to 4 are not shown, but are represented by the respective three dots. Loadings from latent STA variables on the manifest items can vary without any restrictions in a configural invariant model, but the loading of any item is restricted to the same respective value across test administrations when weak invariance is implemented. The free arrows approaching the manifest variables from below represent their respective error terms. Since the same items were applied in every test administration, five item-specific latent variables [93,94] were added to the model to account for indicator specific covariance. Only indicator-specific latent variables for items 1 and 5 are shown (ζ_1_ and ζ_5,_ respectively), but the other three are represented by the three dots in between. The model was identified by setting the factor loading of the first item for every factor to 1.

Longitudinal latent state confirmatory factor analyses (CFA) [92] were used to model the test scores of the figural matrices test (ability-CFA; Figure 4) and the FOF-scale of the FAM (STA-CFA; Figure 5). Since the FAM contained the same questions in every test session, latent variables accounting for indicator-specific covariance [93,94] were included in the STA-CFA. Measurement invariance [95,96] across test sessions for the measurement models of both latent variables was investigated. The most general form of measurement invariance is indicated by configural invariance. In the case of the models employed here, it implies uni-dimensionality of the matrices test and the FOF across test administrations. Weak invariance is a more restrictive form of configural invariance in which factor loadings are kept constant over test sessions. Comparisons between correlations and regressions involving latent variables are valid only when weak invariance holds. Strong invariance is achieved by additionally restricting the item difficulties of a test to be equal across administrations. Note that in the case of the ordinal answer patterns in the matrices test, intercepts of manifest variables are fixed to zero and difficulties are indicated by item category thresholds [94]. Changes in latent variable means across test administrations can be interpreted as true latent variable changes only when strong invariance holds. Since in the described interference reduction approach, differences in latent means over time are only relevant for cognitive ability, strong invariance was targeted for the ability-CFA, whereas weak invariance was targeted for the STA-CFA.

To assess retest as well as interference and deficit effects, the strong invariant ability-CFA and the weak invariant STA-CFA were combined into one LAT model and extended to a full interference model. Note, a neighbor-change model holds the same fit as a strong invariant longitudinal CFA of the same test because the regressions used to introduce the difference variables are residual-free and the degrees of freedom are identical [79]. The measurement invariance equality constraints among both respective measurement models eliminated the problem of rotational indeterminacy [36] and over-identified the model as a whole. However, Halpin et al. [36] derived the AT model and its properties solely for cross-sectional usage. Hence, they did not consider model identification via the implementation of longitudinal measurement invariance constraints. Instead, they suggested imposing testable equality constraints among several interference effects. Therefore, we present an alternative strategy for model identification in Supplementary Materials (Appendix C). Here, a LAT model was used and only configural invariance constraints were implemented. All interference effects of a respective test session were restricted to be equal. This is considered the most conservative approach to interference effect testing [36]. The results delivered by this approach led to the same conclusions.

The interference reduction approach was executed to quantify the amount of interference reduction between all pairs of successive test sessions and to compare interference reduction to retest effect sizes.

### 3.2. Results

In this section, we focus on the results directly related to our research questions. Data, an analysis script, and the results are provided online (see Supplementary Materials) and can be used to assess the descriptive statistics of every measured study variable and the complete parameter estimates of all models.

#### 3.2.1. Descriptive Statistics

Table 1 presents descriptive statistics (means, standard deviations, ranges, internal consistencies, and correlations) for sum scores of the figural matrices test and for the FOF scale. Matrices test scores increased over time, reaching a maximum for the fourth test session and then remaining relatively constant (M_1_ = 7.658; SD_1_ =3.110; M_4_ = 9.938; SD_4_ = 2.621). FOF scores decreased over the entire study length (M_1_ = 16.582; SD_1_ = 6.200; M_7_ = 11.889; SD_7_ = 6.014). Internal consistencies of all measures varied over time but always settled above 0.70 (α = 0.711–0.881).

Only the matrices scores of the first test session was significantly correlated with the FOF scores of any point in time and these correlations were, as expected, negative (range: *r* = −0.133 for FOF at T7 to *r* = −0.197 for FOF at T2). Correlations between scores of a respective test over time were found to be high. For the matrices scores, correlations increased over time, reaching the absolute maximum between the sixth and the last test session (*r* = 0.768). Correlations for the FOF scores followed a similar pattern. Any correlation including a FOF score for a test administration after the second one was above 0.80. The maximum was reached with the correlation between T5 and T6 (*r* = 0.906).

#### 3.2.2. Ability-CFA

Table 2 displays the model fit indices and comparisons for the latent state cognitive ability model in configural, weak, and strong invariant forms. The configural model reached an excellent model fit delivering a non-significant χ^2^ test statistic (χ^2^(3983) = 3283.490, *p* = 1). However, likelihood ratio tests and ΔCFI indicated a significant decline in fit with any further invariance imposition. When strong invariance was implemented, CFI and TLI just barely missed the minimum target value of 0.95 (CFI = TLI = 0.948). The χ^2^-to-df ratio remained under 2.00 (χ^2^/df = 1.627), indicating satisfying model fit. The upper 90% confidence interval bound of the RMSEA settled under 0.08 (RMSEA = 0.053, 90% CI = [0.051, 0.055]). As Chen et al. found in a simulation study, models with such high degrees of freedom are often rejected based on this criterion when estimated with an *N* < 400, even if the model was correctly specified [97]. Thus, we further investigated retest effects on the basis of the strong invariant model and return to this issue in the discussion.

#### 3.2.3. STA-CFA

Table 3 displays the model fit statistics for the configural and weak latent state test anxiety models. The results regarding model fit were mixed for both models. Whereas χ^2^-to-df ratios and RMSEA suggested satisfying model fit, CFI and TLI barely missed their respective thresholds of 0.95 by a maximum margin of 0.02 (TLI for the weak invariant model). Comparison of the models yielded, again, mixed results. Based on the likelihood ratio tests, only configural invariance should be assumed (Δχ^2^(24) = 74.028, *p* < 0.001). The CFI value, on the other hand, decreased by only 0.007, suggesting no substantial decline of model fit when factor loadings are restricted to being equal across test administrations.

#### 3.2.4. Retest Effects

**Figure 6:**
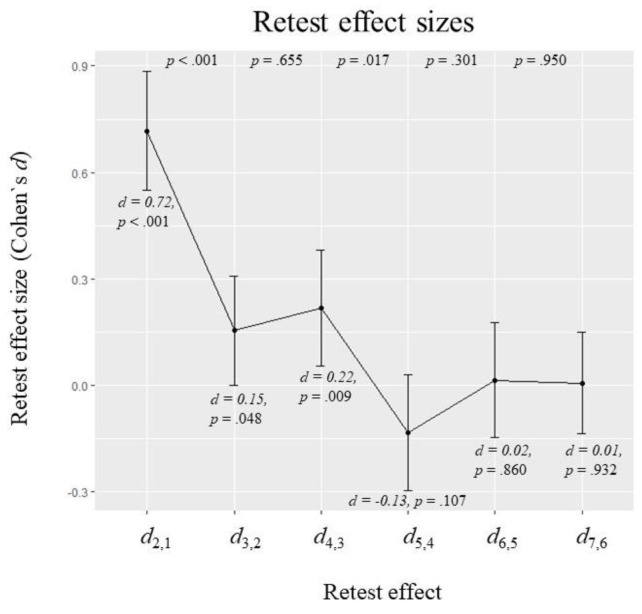
Estimated means of the standardized latent difference variables of the full interference model, which can be interpreted as retest effect sizes in terms on Cohen’s *d* between two successive test administrations. To obtain these parameters, the model was identified by setting the variances of the latent variables to 1. *d*_2,1_ represents the retest effect from the first to the second test administration, etc. Error-bars indicate two-tailed 95% confidence intervals. *p*-values at the top refer to the differences between the respective successive retest effects.

Figure 6 presents the estimated means of the standardized latent difference variables of the full interference model. They can be interpreted as retest effect sizes in terms of Cohen’s *d* between two successive test administrations. The *p*-values at the top indicate whether differences between two successive retest effects were significant. In the more parsimonious models from the interference reduction approach, estimated effect sizes did not change substantially and hypothesis decisions regarding retest effects were identical (see Supplementary Materials).

The largest retest effect was found between the first two test sessions (*d*_2,1_ = 0.72, *p* < 0.001). Retest effects remained positive and significantly different from zero until the fourth test administration (*d*_4,3_ = 0.22, *p* = 0.009). Between the fourth and fifth test session, mean ability actually decreased, but this effect was small and not significant (*d*_5,4_ = −0.13, *p* = 0.107). Compared with the first retest effect, the second decreased substantially (*d*_3,2_−d_2,1_ = −0.56, *p* < 0.001). After that, the only significant change in retest effect size occurred between the third and the fourth retest effect (*d*_5,4_–d_4,3_ = −0.35, *p* = 0.017). However, as already mentioned, no significant change in mean latent ability was observed between the fourth and fifth test administration.

#### 3.2.5. Interference Reduction

Table 4 displays all standardized interference effects of the full interference model in which the interference effects on all items in every test session are estimated. In the first test session, significant interference was found on six ability test items (λ = −0.272 to −0.395). The amount of interference effects decreased by two in the second test session and the overall absolute values of the interference effects decreased (λ = −0.229 to −0.302). In the third test session, only two significant interference effects emerged (λ = −0.274 and λ = −0.290). In every following test session, only one or two of the items were found to be significantly biased due to anxiety. The last row of Table 4 displays item thresholds (i.e., difficulties), which were restricted to be equal across test sessions. The exact order of item difficulties as determined in the calibration study (see Supplementary Materials (Appendix A)) was not replicated, yet item difficulties still roughly increased in the order of presentation. The most and the strongest interference effects were observed on items with intermediate difficulty, although some outliers exist in this regard (e.g., item 1 in the first and item 13 in the second test administration). These results were expected from a certain theoretical viewpoint because anxiety interference reduces the maximum level of the ability at which a person can perform. This reduction will probably not be large enough to hinder the testee from answering particularly easy items correctly and will be irrelevant for the answers to particularly difficult items as the testee would not have been able to solve these in the first place [38,39]. The last column of Table 4 further lists the correlations between latent ability and anxiety for every test session, which are interpreted as deficit effects in the interference reduction approach. No deficits emerged when interference was controlled for. However, a small [80] but significant positive correlation between cognitive ability and STA in the fourth test session was observed (*r* = 0.132, *p* = 0.008). When controlling for interference, these correlations can become positive [36], yet their interpretation is not straightforward. This particular effect might have emerged by chance, as it did not reach significance when an alternative model identification strategy was pursued (see Supplementary Materials (Appendix C)).

Table 5 displays the results of the interference reduction analysis. This more conservative testing procedure delivered results comparable to those derived from Table 4; no significant interference occurred within the last five test sessions. The likelihood ratio test suggested a substantial decline in model fit when interference effects were additionally restricted to zero at the second test administration (Δχ^2^(13) = 24.432, *p* = 0.027). However, the model CFI only reduced by 0.006 in that case. Results unambiguously suggested a decreased model fit when interference effects were additionally assumed to be absent at the first test session (Δχ^2^(13) = 46.045, *p* < 0.001, ΔCFI = 0.014).

## 4. Discussion

In this study, we extended a structural equation model specifically designed to test the interference and deficit hypotheses on the anxiety test performance relationship for use in longitudinal studies. This model allows the investigation of the connection of test anxiety to test performance and to changes in test performance. It was applied in an empirical study where we explored retest effects occurring when taking a figural matrices test seven times. Test performance improved up to the fourth test session before plateauing. The gain in test performance was the largest between the first and second test session and leveled off with increasing test repetitions. These findings are in line with the power law of practice [98]. The results suggested anxiety interferences to be the cause of impaired test performance. No significant negative correlation between ability and anxiety emerged when these interferences were controlled for. The amount and magnitude of interference effects decreased across test administrations. A substantial reduction in interference between the first two test administrations was aligned with the largest retest effect. A smaller retest effect in the third test session occurred with a smaller reduction in interference. Reduced interference did not explain the last observed mean ability estimate increase. Therefore, a reduction in anxiety-induced measurement bias cannot completely explain the emersion and development of retest effects. We recommend to refer back to the theoretical framework reported by Lievens et al. [22] and explore the role of other potential factors in future longitudinal studies.

### 4.1. Implications and Future Research

Before any further theoretical deliberations on retest effects and test anxiety, we first discuss the statistical model itself, as it should not be viewed without criticism. As Halpin et al. [36] acknowledged, imposing equality constraints upon the interference effects for model identification in cross-sectional settings is not an ideal solution because all interferences can be different in the true model. We bypassed this issue by identifying the model with the implementation of longitudinal invariance constraints upon ability and anxiety measurements. However, these strong assumptions might also be violated in the true model. In this regard, our results were ambiguous (see the last section of the discussion). Nevertheless, we replicated evidence of an overall absence of deficit effects and a presence of substantial interference in the first two test sessions using a configural invariant LAT model (see Supplementary Materials (Appendix C)). In that model, we implemented a different model identification strategy that resembles the original approach, as proposed by Halpin et al. This raises the question: Is the methodological framework robust against certain assumption violations yet sensitive to others? Simulation studies can provide further insight into this matter. Systematic variations in the study parameters should include the sizes of interference and deficit effects (zero for both included), (in)equality of interference effects, and forms of (partial) measurement invariance. In longitudinal settings, the respective inter-correlations of anxiety and ability states (or latent difference variables, depending on whether the LAT or the full interference model is used) represent parameters that are not present in the original AT model. Halpin et al. demonstrated that in cross-sectional settings, the partial correlation between latent ability and a third latent variable (e.g., a different cognitive ability) controlled for anxiety does not depend on the identification constraints. Our proposed models, however, do not statistically represent that case. Firstly, latent ability in every test session was controlled for by a different latent anxiety state variable. Secondly, latent anxiety states were modelled to correlate with each other. Thus, these latent correlations added to the original AT model might have important implications for type I and type II errors in interference and deficit detection given certain model identification strategies. We strongly recommend considering these issues first before providing suggestions regarding the control for anxiety in personnel selection settings to practitioners.

The plateau of the mean ability estimate after the fourth test session seems to be at odds with the meta-analytic results reported by Scharfen et al. [6], where retest effects of fluid reasoning tasks (often measured using figural matrices tests) were observed only up to the third test session. However, as Scharfen et al. [5] found, retest effects in the domain of working memory regularly remain up to the fourth test session. The distinction between reasoning and working memory has been heavily discussed and high correlations between measurements of both constructs are consistently found (e.g., [99,100,101]). This is something to consider, especially in cases where the rules of item object patterns are explained to testees beforehand, such as in the present study. In these cases, the known rules have to be actively remembered, whereas representations of the item objects have to be actively manipulated in working memory to test the fit of object patterns to a rule.

We see potential in the role of working memory to clarify mixed findings on the presence of anxiety-induced measurement bias. Sommer and Arendasy found no evidence for the interference hypothesis in three consecutive studies [38,56,65]. They also analyzed their data via the AT model (or a variant with a manifest instead of a latent anxiety variable), showing explanations for result heterogeneity due to methodological differences are unlikely. One of these studies employed a knowledge-based multiple choice test [56]. The other two covered a wide range of cognitive abilities by investigating several tests [38,65]. Although one of these presented rule-based numerical reasoning tasks, whether any of these tests reached the same amount of working memory load imposed by the complex figural patterns present in the current study is questionable. In accordance, Ng and Lee [46] found anxiety-induced measurement bias specifically at higher levels of working memory load. Neuroscientific evidence shows that state anxiety (as opposed to trait anxiety) increases brain activity in the amygdala and other areas associated with bottom-up threat detection [47,59,60,102]. On the basis of attention control theory [59], the hypothesis that this depletes or inhibits cognitive functions for top-down processing required in working memory tasks could be examined.

The distinction between state and trait test anxiety is another important aspect for consideration. In the current study, we focused on situational (i.e., state) test anxiety because the interference reduction approach implies a habituation to the test situation. FOF sum score correlations, however, indicated very high state stabilities (Table 1) and latent state inter-correlations supported this finding (see Supplementary Materials). The high stability of a construct does not necessarily imply stability of the constructs effect on other variables. In this regard, future research on the interference reduction approach should consider trait anxiety. However, we propose that interference effects from trait test anxiety will not emerge, at least not if state test anxiety is controlled for. Based on our deliberations above, interference seems to be specific to an enhanced state of anxiety and to have a negative impact specifically on working memory capacity, whereas deficits are more related to general trait anxiety [56,65] and hence will be observed with tasks with lower working memory capacity demands. Thus, future research should focus on the differentiation between state and trait test anxiety and their relationship to the cognitive load demands of different cognitive ability tests.

Future studies should also apply frequent assessments of test anxiety within a single test session. It seems reasonable to assume that the increase in test anxiety is due to a perceived discrepancy between the difficulty of a given task and one’s own cognitive ability. This means that fluctuations in test anxiety across the different items of a test are to be expected. Measuring item-specific anxiety can lead to refinements in the deficit hypothesis, because it allows testing whether anxiety emerges specifically in situations where a large discrepancy between item difficulty and cognitive ability is observed. This finding would be in line with the deficit hypothesis because a large discrepancy of that kind would mean that the probability of solving the item correctly is very low, regardless of the experienced anxiety level. To control for interferences, a general anxiety factor could be aggregated from the item-specific anxiety values. Item-specific assessment of anxiety via a questionnaire would be aggravating for study participants, but valid one-question anxiety scales are available [103].

Potential confounds of test anxiety that might be especially influential in the early test sessions should be considered. Comprehension of the test is an important aspect to evaluate in that context. Testees might consider themselves capable of solving the test items in general, but might fear that the test outcome will not be representative of their true ability when the structure of the tasks is not yet fully understood. It is this fear that eventually induces measurement bias. This could especially apply to tests with beforehand rule explanations. Future studies on the interference reduction approach should hence control for incomprehension.

Other important construct-irrelevant factors to be considered in the context of the current study are perceived challenge, interest, and probability of success—the other facets of current achievement motivation [52]. CAM and test performance are positively correlated [49], but theoretical and statistical specifications on how these individual facets contribute to this correlation are not as detailed as for FOF. It is not even clear whether any of these facets can causally influence test performance like FOF in the context of the interference hypothesis. Their relationship to test performance changes is not straightforward, either. In what way should what influence of perceived challenge on test performance change with multiple test administrations? Should we expect a change in perceived challenge to begin with? Similar questions arise for interest. We could argue that multiple measurement occasions increase the familiarity with the test procedure so that it seems more approachable and, hence, the interest of testees is increased. Would this then lead to an increased test performance? If so, is it the familiarity or the interest that positively impacts the (re)test score? From an opposing viewpoint, we could argue that repeated assessments of intelligence would simply bore testees and hence decrease their interest. The facets of perceived challenge and interest and their relation to retest effects may be best assessed via experimental manipulations and not via mere measurement repetitions. Regarding probability of success, change due to repeated testing does not seem implausible, especially when feedback on test performance is provided. People with certain personality traits (supposedly rather agreeable or neurotic testees) might underestimate their probability of success at the beginning when they are confronted with the often rather abstract test material. This might have a discouraging effect and hinder them from performing to their true potential. With repeated positive feedback on test items they might, however, more realistically estimate their ability and achieve higher scores. In other words, an underestimation of one’s probability of success leads to an increased emotional state of discouragement, which in turn induces systematic negative measurement bias on ability test items. These effects decrease in size with increasing test repetitions. An extended and modified full interference model could represent this theory. Post-hoc analyses of our data with multiple *t*-tests revealed an increase in probability of success up to the fourth test administration before reaching a plateau. Perceived challenge and interest did not fluctuate in a recognizable systematic pattern.

### 4.2. Limitations and Future Research

With regards to issues of construct validity, a methodological drawback of this study must be discussed. Explaining the rules of a reasoning test beforehand has the advantage of preventing multiple solutions to items because the cognitive operations required to deduce the correct answers are determined by the rules. The employment of alternative possible rules leading to different conclusions that were not considered by the test creators is hence invalid by definition. The major disadvantage, however, is that this arguably changes the construct to be measured. Important hallmarks of fluid reasoning are operations of inductive and deductive reasoning to solve novel problems [104]. Cattell explicitly stated that for a measure of fluid reasoning, a testee must not have “recourse to answers to such complex issues already stored in memory” [105] (p. 115). Thus, whereas MatrixDeveloper test items definitely require inductive and deductive reasoning processes for solving, it is questionable how well these items represent a measure of fluid reasoning and a proxy measure for *g* accordingly when the rules are known beforehand (see also our deliberations on working memory in the previous section). We therefore recommend the usage of our proposed SEM in longitudinal studies with diverse ability measures.

A shortcoming of the current study lies within its setting. In contrast with assessment centers, where low test performance can result in undesired outcomes, such as being excluded from consideration for job positions, here, participants faced no negative consequences for poor performance. Also, test anxiety was not experimentally manipulated between test sessions or groups. This, of course, limits the intensity of potentially experienced test anxiety as solely intrinsic values, like competence-based self-confidence, are at risk. Participants were informed that the employed matrices test did not undergo a normalization process. Received feedback on the test items therefore limited interpretations of between-person comparisons. Study replications in high-stakes settings could increase the ecological validity of our findings [65].

The lack of a high-stakes setting and experimental manipulations of test anxiety also in part explain the previously mentioned high stability of anxiety states across test sessions. This high state stability is undesirable in longitudinal studies because it implies multi-collinearity of latent variables. Similar considerations arise for ability test items. To control for ceiling effects, we had to exclude many participants (*N* = 51), which suggests that the tests were generally too easy. This was a surprise to us since participants in the calibration study were also recruited amongst university students and academics. This result can probably in part be explained by motivation being lower for participants in the calibration study as they did not receive monetary remuneration and hence were less eager to perform their best. Another likely reason is the calibration study being cross-sectional and hence item difficulties were probably overestimated due to unfamiliarity with the abstract test material. Presenting easy items repeatedly can also lead to linear dependencies of multiple variables across the test administrations, producing suboptimal conditions for the statistical analysis. However, these are less severe when direct relationships between latent state variables are modeled by correlations instead of multiple regressions, which is the case in all of the applied models. Although we encountered multi-collinearities between latent variables, all estimation algorithms converged normally and no unexpected or uninterpretable parameter estimates or standard errors were produced (see Supplementary Materials). Nevertheless, the utility of optimal study design to avoid such conditions should be stressed. Ideally, researchers would select the participants based on their abilities so that enough variance on every test item is produced. This requires pre-study knowledge of participants’ ability parameters, which are provided only in rare occasions. Alternatively, test items should be carefully chosen, so that their difficulty range sufficiently covers the expected range in ability while simultaneously considering a decrease in test difficulty over time.

Despite these caveats, we were able to demonstrate the overall stability of our findings. We produced comparable results regarding retest effect development, interference reduction, and the lack of observed deficits with the complete sample including ceiling effects and altered modeling approaches (Supplementary Materials (Appendices B–D)).

### 4.3. Deliberations on Measurement Invariance in Multiple Test Administrations

Lastly, we address a general and major problem in studies with multiple test administrations—measurement invariance of applied test procedures. In our study, we provided seven different cognitive ability tests that were assumed to be parallel. In other words, whereas all items differed from each other, all seven tests had the same difficulty and the difficulty of every item from each test session matched the difficulty in every other test session. This was achieved by selecting items based on their difficulty parameters estimated in a calibration study (see Supplementary Materials (Appendix A)).

The CFA results on the matrices test revealed that only configural invariance should be assumed, meaning that item loadings and difficulties differed across test sessions. Assuming strong invariance regardless and testing retest effects in the ability change model framework under such circumstances has important implications for the interpretation of model retest effect parameters. Although an alternative name for this model is true change model [78], these true changes do not refer to the theoretically assumed cognitive ability when strong invariance does not hold. However, the alternative and classic assessment of retest effects, computation of test sum score differences, is worse because this procedure implies the more restrictive strict measurement invariance [95,96]. Henceforth, the ability change model is the best of the two options. Researchers should keep in mind that these latent differences do not necessarily reflect actual improvements in cognitive ability.

The more difficult problem arises when considering potential explanations for the lack of measurement invariance; one possible explanation is also an explanation for retest effects, i.e., the research topic of interest. In the following, we report a method of addressing this problem (for similar considerations see [33]).

Employing the exact same test for every test session should be avoided, especially when feedback is provided on item answers because retest effects could then be explained by simple memory effects of previously encountered items. This implies a loss of construct validity in later test sessions [22]. Yet, the literature suggests that retest effects also occur when parallel forms of tests are provided [4,5,6]. Accordingly, attempts should be made to create and use different but parallel test versions. When results suggest that these also cannot be assumed to hold measurement invariance, questions emerge: Were the pre-study estimations of difficulty parameters biased or inaccurate by chance? Did we determine invariance of our parallel test forms in a population different from our study population? Does the measurement of our latent ability construct actually change over time? The last question asks for explanations of retest effects that jeopardize construct validity at earlier or later test sessions, as suggested by Lievens et al. [22]. The accuracy of one explanation cannot be checked if all other explanations are not controlled, which is a difficult, if not impossible, task. Returning to the current study, the overall fit of the strong invariant cognitive ability model was still acceptable. This corroborates our selection of presumably parallel items. However, the significant decrease in model fit, when compared with less restrictive models, cannot be ignored. The whole idea of the interference reduction approach can be formulated as a longitudinal change in ability measurement (implying inequality of factor loadings and item difficulties) based on changes of systematically induced measurement bias. Testing this approach thereby becomes obsolete when any form of invariance more restrictive than configural can be seen as given. The results of the approach itself suggested that a lack of cognitive ability measurement invariance could partially be explained by anxiety-induced measurement bias in earlier test sessions.

## 5. Conclusions

In this paper, we presented a structural equation model that allows for an investigation of the interference and deficit hypotheses on the negative cognitive ability test anxiety relationship in longitudinal studies. The model further allows the comparison of retest effect sizes with the magnitude of interference reduction across the test administrations. In a first study incorporating this approach, we found that retest effects reflect a reduction in anxiety-induced measurement bias, at least to a certain extent. Using the employed figural matrices tests, retest effects were observed up to the fourth test session, where they plateaued. Situational test anxiety produced substantial measurement bias on test items in the first two test sessions. This suggests an increase in the reliability and validity of cognitive ability measurement within the first test repetitions. Yet, we also found that that the interference hypothesis cannot completely explain the retest effect phenomenon. Future research should focus on the statistical properties of the model. Differences between trait and state anxiety, differences between cognitive abilities, the cognitive working memory load imposed by specific tests, and other potential predictors of retest effects require further investigations.

## Figures and Tables

**Table 1:** Descriptive statistics and correlations of study variable sum scores.

		Descriptive Statistics				Correlations													
						FM							FOF						
Measure	Test session	Mean	SD	Min	Max	1	2	3	4	5	6	7	1	2	3	4	5	6	7
FM	1	7.658	3.11	1	13	0.776													
	2	9.187	2.63	0	13	0.700 ***	0.711												
	3	9.631	2.69	0	13	0.640 ***	0.684 ***	0.761											
	4	9.938	2.621	1	13	0.584 ***	0.705 ***	0.660 ***	0.754										
	5	9.782	3.043	0	13	0.579 ***	0.616 ***	0.738 ***	0.687 ***	0.819									
	6	9.791	3.058	0	13	0.619 ***	0.690 ***	0.695 ***	0.681 ***	0.714 ***	0.819								
	7	9.822	2.905	0	13	0.594 ***	0.639 ***	0.707 ***	0.643 ***	0.736 ***	0.768 ***	0.798							
FOF	1	16.582	6.2	5	31	−0.157 *	−0.104	−0.088	−0.05	−0.042	−0.067	−0.054	0.84						
	2	15.116	6.352	5	34	−0.197 **	−0.094	−0.087	−0.032	0.023	−0.033	−0.037	0.785 ***	0.881					
	3	13.569	6.001	5	32	−0.177 **	−0.131	−0.099	−0.062	0.005	−0.033	−0.057	0.729 ***	0.864 ***	0.868				
	4	12.929	5.95	5	28	−0.148 *	−0.058	−0.04	−0.035	0.02	−0.013	−0.002	0.700 ***	0.826 ***	0.888 ***	0.867			
	5	12.48	6.15	5	28	−0.148 *	−0.11	−0.085	−0.1	−0.013	−0.011	−0.014	0.633 ***	0.812 ***	0.882 ***	0.891 ***	0.878		
	6	12.36	6.005	5	30	−0.159 *	−0.124	−0.107	−0.116	−0.068	−0.069	−0.077	0.574 ***	0.759 ***	0.826 ***	0.838 ***	.906 ***	0.875	
	7	11.889	6.014	5	28	−0.133 *	−0.106	−0.057	−0.072	−0.013	−0.057	−0.04	0.594 ***	0.749 ***	0.851 ***	0.853 ***	0.861 ***	0.864 ***	0.877

Notes. *N* = 225. SD = Standard Deviation; Min = Minimum; Max = Maximum; FM = Figural Matrices; FOF = fear-of-failure scale of the FAM. The diagonal of the correlation matrix presents coefficients of internal consistency for the respective measure at a given test session. For the matrices test, this is given by the Kuder Richardson coefficient (Formula 20) for binary data, and by Cronbach’s α for the FOF scale. * *p* < 0.05; ** *p* < 0.01; *** *p* < 0.001.

**Table 2:** Model fit and comparisons of the configural, weak, and strong invariant ability-CFA.

Implemented Invariance	Δχ^2^ (df)	*p*	χ^2^ (df)	*p*	χ^2^/df	RMSEA [90% CI]	CFI	TLI
Configural	-	-	3283.490 (3983)	1	0.824	0.000 [0.000, 0.000]	1.000	1.000
Weak	168.960 (72)	<0.001	6038.581 (4055)	<0.001	1.489	0.047 [0.044, 0.049]	0.960	0.960
Strong	727.390 (71)	<0.001	6712.612 (4126)	<0.001	1.627	0.053 [0.051, 0.055]	0.948	0.948

Notes. df = degrees of freedom; RMSEA = Root mean square error of approximation; CI = Confidence interval; CFI = Comparative Fit index; TLI = Tucker–Lewis index. Models were identified by setting the factor loading of the first matrices item of any test session to 1.

**Table 3:** Model fit and comparisons of the configural and weak invariant STA-CFA.

Implemented Invariance	Δχ^2^ (df)	*p*	χ^2^ (df)	*p*	χ^2^/df	RMSEA [90% CI]	CFI	TLI
Configural	-	-	845.657 (504)	<0.001	1.678	0.055 [0.049, 0.061]	0.945	0.935
Weak	74.082 (24)	<0.001	913.722 (528)	<0.001	1.731	0.057 [0.051, 0.063]	0.938	0.930

Notes. df = degrees of freedom; RMSEA = Root mean square error of approximation; CI = Confidence interval; CFI = Comparative fit index; TLI = Tucker–Lewis index. Models were identified by setting the factor loading of the first item for every factor to 1.

**Table 4:** Standardized interference effects and correlations of latent ability and anxiety (deficit effects) of the full interference model.

Test Session					Item									*r*_η,ξ_
	1	2	3	4	5	6	7	8	9	10	11	12	13	
1	−0.300 **	0.088	−0.272 *	0.041	−0.095	−0.377 ***	−0.347 ***	−0.340 ***	−0.159	−0.395 ***	−0.188	−0.106	−0.116	−0.060
2	−0.041	0.064	−0.302 **	−0.074	−0.168	−0.014	−0.037	−0.014	−0.254 **	−0.236 **	−0.127	0.022	−0.229 **	0.134
3	−0.039	−0.178	−0.274 *	−0.118	−0.129	−0.137	−0.290 **	−0.153	−0.087	0.065	0.032	−0.071	0.021	−0.006
4	−0.039	−0.170	−0.196	−0.094	−0.042	−0.246 *	0.026	−0.339 **	−0.133	−0.041	−0.038	0.120	−0.166	0.132 **
5	−0.109	−0.049	−0.21	−0.100	−0.065	−0.012	−0.037	−0.003	−0.195 *	−0.024	0.053	0.067	0.047	0.031
6	−0.194	0.016	−0.078	0.009	0.052	−0.237 *	−0.168	0.002	−0.037	−0.007	−0.148	−0.015	−0.021	−0.032
7	−0.046	0.117	−0.188	0.034	−0.215 *	0.010	0.017	−0.048	−0.315 **	−0.144	−0.078	0.059	−0.086	−0.035
Threshold	−1.019	−0.933	−1.062	−0.702	−0.760	−0.821	−0.536	−0.493	−0.549	−0.447	−0.248	0.059	0.025	

Notes: *N* = 225; *r*_η,ξ_ = Correlation between latent ability and latent anxiety. Thresholds reflect item difficulties, which were restricted to be equal across test sessions. The model was identified by setting the variances of the latent variables to 1. Significant interference effects are printed in bold. * *p* < 0.05; ** *p* < 0.01; *** *p* < 0.001.

**Table 5:** Model fit and comparisons of nested models of interference effects in the interference-reduction approach.

Test Sessions with Modeled Interference Effects	Δχ^2^ (df)	*p*	χ^2^ (df)	*p*	χ^2^/df	RMSEA (90% CI)	CFI	TLI
1 to 7	-	-	9766.433 (7753)	<0.001	1.230	0.034 [0.032, 0.036]	0.971	0.971
1 to 6	16.882 (13)	0.205	10,079.649 (7766)	<0.001	1.300	0.036 [0.034, 0.038]	0.967	0.966
1 to 5	11.459 (13)	0.572	10,272.688 (7779)	<0.001	1.321	0.038 [0.036, 0.040]	0.964	0.964
1 to 4	9.749 (13)	0.714	10,423.506 (7792)	<0.001	1.338	0.039 [0.037, 0.041]	0.962	0.962
1 to 3	20.410 (13)	0.085	10,790.511 (7805)	<0.001	1.383	0.041 [0.039, 0.043]	0.957	0.957
1 and 2	18.128 (13)	0.153	11,126.464 (7818)	<0.001	1.423	0.043 [0.042, 0.045]	0.952	0.952
1	24.432 (13)	0.027	11,581.707 (7831)	<0.001	1.479	0.046 [0.044, 0.048]	0.946	0.946
None	46.045 (13)	<0.001	12,525.000 (7844)	<0.001	1.597	0.052 [0.050, 0.053]	0.932	0.932

Notes. df = degrees of freedom; RMSEA = Root mean square error of approximation; CI = Confidence interval; CFI = Comparative fit index; TLI = Tucker–Lewis index. Models were identified by setting the variances of latent variables to 1.

## Supplementary Materials

The following are available online at (https://www.mdpi.com/2079-3200/7/4/22/s1): “Supplementary_Appendices.pdf” (see this document for the appendices mentioned throughout the article); “Data.rda”; “analysis_script.R”; “analysis_results.txt”.
