# Supplementary material for: The Impact of Situational Test Anxiety on Retest Effects in Cognitive Ability Testing: A Structural Equation Modeling Approach
Source: J Intell. 2019 Sep 23;7(4):22. doi: 10.3390/jintelligence7040022 (PMC6963292; doi:10.3390/jintelligence7040022)
Supplement: Supplementary file 1 [file jintelligence-07-00022-s001.zip › Supplementary_Appendices.pdf]

The impact of situational test anxiety on retest effects in cognitive ability testing: A structural equation modeling approach – Appendices

Correspondence: David Jendryczko (david.jendryczko@uni-konstanz.de)

Appendix A

Pre-study item easiness parameters

From a previous calibration study for the automatic item generation software *MatrixDeveloper* [1] executed in the department, easiness parameters (probability of solving an item correctly given average ability) of 180 different items were available prior to study begin. Out of this pool, 93 items were selected for the study via a matching system. We considered an ability test with thirteen items per test session appropriate to sufficiently cover a wide range in cognitive ability. Hence, we chose thirteen items of increasing difficulty for the first test session and picked six items for each of these as an equivalent for the remaining test sessions to create seven parallel test forms with nearly identical difficulty. Table A1 displays the item easiness parameters of all study matrices items as well as easiness mean and standard deviation for every test session.

Figure A1 presents boxplots of item easiness parameter distributions for every test session. The figure suggests homoscedasticity of easiness parameters across all sessions. This was further supported by a Bartlett ($K^2(6) = 0.201, p = 1$), a Levene ($F(6, 84) = 0.104, p = .995$) and a Fligner-Killeen ($\chi^2(6) = 0.470, p = .998$) test. An ANOVA revealed no differences in mean easiness across sessions ($F(6, 84) = 0.001, p = 1$).

Table A1. Easiness-parameters of matrices test items as estimated pre-study.

Test session	Item													Mean	SD
	1	2	3	4	5	6	7	8	9	10	11	12	13		
1	.929	.844	.810	.794	.751	.700	.556	.431	.400	.383	.300	.252	.052	.554	.271
2	.956	.828	.787	.754	.707	.707	.555	.441	.428	.408	.283	.252	.055	.551	.265
3	.907	.861	.773	.773	.724	.686	.591	.441	.413	.390	.313	.244	.092	.554	.257
4	.907	.812	.810	.789	.712	.667	.597	.494	.404	.354	.309	.226	.097	.552	.257
5	.916	.765	.748	.748	.726	.661	.627	.518	.476	.424	.324	.216	.100	.558	.241
6	.886	.871	.806	.797	.724	.712	.551	.477	.452	.332	.311	.201	.100	.555	.265
7	.935	.823	.822	.802	.724	.616	.541	.473	.447	.343	.322	.256	.070	.552	.262

Notes. $N = 509$. SD = Standard Deviation. Easiness refers to the probability of solving an item correctly given average ability.

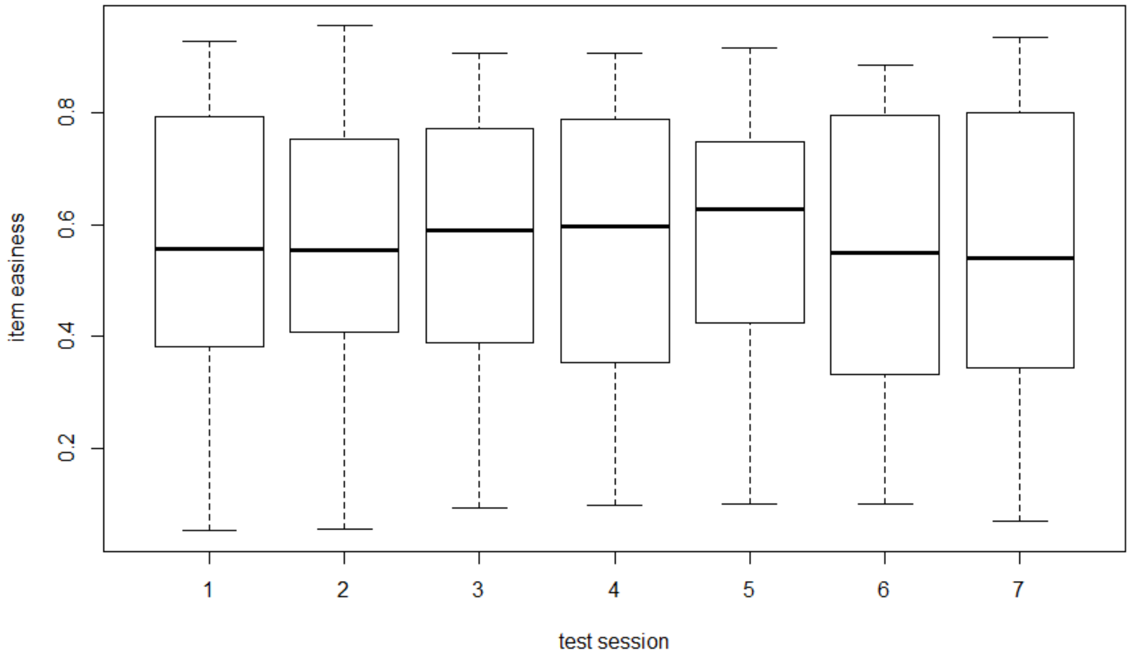

Figure A1. Boxplots of item easiness distribution for every test session. Item easiness is to be interpreted as probability of solving an item correctly given average ability.

Appendix B

Results for the complete sample including participants with ceiling effects

In this section we provide analysis results for the complete sample in which no participants with ceiling effects were excluded (*N* = 276). In this sample, 25.4% reported to be male, 74.3% reported to be female (one missing value), the mean age was 23.29 years (*SD* = 4.56), 48.19% studied psychology, 7.61% studied economics, 2.17% studied communications, and 9.42% were not students.

Table B1 displays descriptive statistics and correlations of matrices test and fear of failure sum scores for each test session.

Table B1. Descriptive statistics and correlations of study variable sum scores for the complete sample.

Descriptive statistics						Correlations													
						FM							FOF						
Measure	Test session	Mean	SD	Min	Max	1	2	3	4	5	6	7	1	2	3	4	5	6	7
FM	1	8.144	3.007	1	13	.777													
	2	9.630	2.616	0	13	.714***	.729												
	3	10.138	2.678	0	13	.661***	.715***	.785											
	4	10.409	2.590	1	13	.613***	.723***	.700***	.779										
	5	10.261	2.949	0	13	.600***	.646***	.753***	.718***	.830									
	6	10.272	2.991	0	13	.624***	.696***	.716***	.699***	.729***	.834								
	7	10.279	2.817	0	13	.623***	.659***	.731***	.675***	.753***	.777***	.808							
FOF	1	16.739	6.132	5	35	-.133*	-.083	-.055	-.021	-.013	-.034	-.027	.842						
	2	15.395	6.227	5	34	-.151*	-.049	-.034	.008	.058	.006	.009	.789***	.876					
	3	13.949	6.009	5	33	-.122*	-.062	-.022	.001	.056	.024	.003	.741***	.869***	.869				
	4	13.199	5.895	5	32	-.097	-.017	.005	.005	.056	.029	.041	.712***	.826***	.884***	.866			
	5	12.703	5.996	5	29	-.108	-.078	-.043	-.059	.022	.029	.021	.652***	.800***	.867***	.888***	.873		
	6	12.580	5.932	5	32	-.109	-.083	-.058	-.069	-.029	-.031	-.030	.591***	.755***	.820***	.841***	.899***	.872	
	7	12.196	5.890	5	32	-.074	-.049	-.001	-.022	.027	-.006	.010	.609***	.749***	.841***	.854***	.858***	.869***	.873

Notes. N = 276. SD = Standard Deviation; Min = Minimum; Max = Maximum; FM = Figural Matrices; FOF = Fear of Failure scale of the FAM. The diagonal of the correlation matrix presents coefficients of internal consistency for the respective measure at a given test session. For the matrices test, this is given by the Kuder Richardson coefficient (formula 20) for binary data and for the Fear of Failure scale it is given by Chronbach's α.
*p < .05; **p < .01; ***p < .001

Table B2 displays fit indices for the configural, weak and strong invariant cognitive ability-CFA. As in the main study, results indicated substantial decline in model fit when any invariance restrictions were imposed on the model. However, we received satisfactory fit for a strong invariant model with regards to most indices.

Table B2. Model fit and comparisons of the configural, weak and strong invariant ability-CFA estimated within the complete sample (N = 276).

Implemented invariance	$\Delta\chi^2(df)$	p	$\chi^2(df)$	p	χ^2/df	RMSEA [90%CI]	CFI	TLI
Configural	-	-	3193.936 (3983)	1	0.802	.000 [.000, .000]	1.000	1.000
Weak	173.470 (72)	< .001	6158.367 (4055)	< .001	1.519	.043 [.041, .046]	.969	.969
Strong	780.810 (71)	< .001	6857.222 (4126)	< .001	1.662	.049 [.047, .051]	.960	.960

Notes. df = degrees of freedom; RMSEA = Root Mean Square Error of Approximation; CI = Confidence Interval; CFI = Comparative Fit Index; TLI = Tucker-Lewis Index. Models were identified by setting the factor loading of the first matrices item of any test session to 1.

We encountered a problem when estimating model parameters for the STA-CFA with the data set including the participants with ceiling effects. The estimated residual variances of some manifest variables were negative (Heywood-cases). Parameter estimates for this model with the main study sample revealed that variance and factor loadings for the indicator specific latent variable for the second FOF item were not significantly different from zero (see the analysis script from the supplementary material). This suggests that inter-correlations for this item across test sessions can be traced back to the latent anxiety states, and item specific characteristics do not further contribute to covariance explanation.

We constrained the variance of this latent indicator specific variable to the value that was estimated with the main study sample (σ^2 = 0.05). This eliminated the problem of Heywood-cases. Table B3 displays fit statistics and comparisons for this model in configural and weak invariant form. Results regarding model fit did not change substantially when the above mentioned variance was estimated freely (and hence Heywood-cases were accepted) or the indicator specific latent variable for the second FOF item was dismissed completely (see the analysis script). Fit decreased substantially when weak invariance was imposed, yet was overall still satisfactory with regards to χ^2-df-ratio and RMSEA. Conclusions for the interference-reduction approach did not depend on measurement invariance restrictions for this model (see Appendix C).

Table B3. Model fit and comparisons of the configural and weak invariant STA-CFA estimated within the complete sample (N = 276).

Implemented invariance	$\Delta\chi^2(df)$	p	$\chi^2(df)$	p	χ^2/df	RMSEA [90%CI]	CFI	TLI
Configural	-	-	886.265 (505)	< .001	1.755	.052 [.047, .057]	.950	.941
Weak	499.22 (24)	< .001	989.831 (529)	< .001	1.871	.056 [.051, .061]	.939	.932

Notes. df = degrees of freedom; RMSEA = Root Mean Square Error of Approximation; CI = Confidence Interval; CFI = Comparative Fit Index; TLI = Tucker-Lewis Index. Models were identified by setting the factor loading of the first item for every factor to 1.

Figure B1 presents estimated means of the standardized latent difference variables of the full interference model. While the exact numerical values differed slightly from the reduced sample, the pattern of results and hypothesis-decisions remained exactly the same.

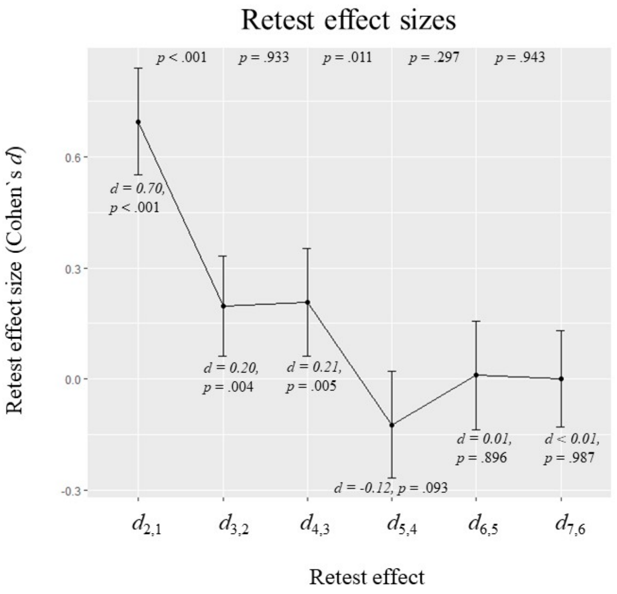

Figure B1. Means of the standardized latent difference variables of the full interference model estimated within the complete sample (N = 276). They can be interpreted as retest effect sizes in terms on Cohen`s d between two successive test administrations. To obtain these parameters the model was identified by setting the variances of the latent variables to 1. $d_{2,1}$ represents the retest effect from the first to the second test administration. The remaining d are to be understood accordingly. Error-bars indicate two-tailed 95%-confidence intervals. p-values at the top relate to the difference between the respective successive retest effects.

Table B4 displays all interference effects of the full interference model. Similar to the results presented in the main study most interference effects were found at the first test session. Here, measurement of five items was biased due to test anxiety. From the second to the fourth test session, only one or two interference effects were found. After that, no more interference took place, with the exception of the last test session. Here, measurement of one item was significantly biased. The last row of the table displays item thresholds (i.e. difficulties). The most, and the strongest interference effects occurred on items with intermediate difficulty. The last column of the table displays the correlations of latent ability and latent anxiety for every test session, which can be interpreted as deficit parameters. No deficits were found after controlling for interference effects.

Table B4. Standardized interference effects and correlations of latent ability and anxiety (deficit-effects) of the full interference model.

Testsession	Item													$r_{\eta,\xi}$
	1	2	3	4	5	6	7	8	9	10	11	12	13	
1	**-0.256***	0.047	-0.217	0.026	-0.024	**-0.296****	-0.227*	**-0.234****	-0.124	**-0.270****	-0.148	-0.105	-0.027	-.071
2	-0.013	0.047	**-0.240***	-0.069	-0.122	-0.025	-0.008	0.035	**-0.231****	-0.156	-0.085	0.054	-0.124	.087
3	-0.009	-0.127	**-0.220***	-0.083	-0.083	-0.115	-0.197	-0.100	-0.048	0.081	0.082	-0.026	0.073	.027
4	-0.020	-0.129	-0.144	-0.020	0.013	**-0.208***	0.021	**-0.207***	-0.089	-0.032	-0.012	0.136	-0.099	.097*
5	-0.102	-0.017	-0.076	-0.048	-0.037	0.048	-0.026	0.033	-0.128	0.039	0.090	0.135	0.083	-.004
6	-0.133	0.077	-0.055	0.072	-0.011	-0.169	-0.086	0.076	0.000	0.055	-0.087	0.047	0.046	-.085
7	-0.007	0.149	-0.113	0.081	-0.179	0.042	0.070	0.027	**-0.269****	-0.062	-0.045	0.101	-0.014	.005
Threshold	-1.154	-1.056	-1.200	-0.834	-0.892	-0.958	-0.666	-0.649	-0.708	-0.602	-0.397	-0.116	-0.161	

Notes. N = 276. $r_{\eta,\xi}$ = Correlation between latent ability and latent anxiety. Thresholds reflect item difficulties. They were restricted to be equal across test sessions. The model was identified by setting the variances of the latent variables to 1. Significant interference effects are printed in bold.
*p < .05; **p < .01

Table B5 displays model comparisons of nested models in the interference-reduction approach. Based on this, significant interference occurred only at the first test session, which stands in contrast to the main study results where substantial interference happened at the first two test administrations.

This probably reflects a drawback of the study that has been mentioned in the discussion of the main article: the study took place in a low-stake setting. Due to the participants' subdued anxiety experience, the power to find any potential interference effects was decreased. This power is, of course, even more limited when participants that did exceptionally well on the ability tests are included in the analysis. Moreover, a comparison between Table 1 and Table B1 reveals that mean FOF sum scores at every test session increase when participants with ceiling effects are included. In other words, some of the highest scoring participants had the highest anxiety values. Two-sample Welch-tests of FOF sum scores between the main study sample and the participants with ceiling effects further support this finding (see Table B6). For these high achieving participants, potential interference effects could not have been detected. Ability items were too easy for them to begin with, so that they were able to solve them correctly regardless of their level of experienced anxiety. This in turn blurs any existing interferences in the complete sample for inferential statistical detection. Nevertheless, even in that case, evidence for anxiety-induced measurement bias at the earliest test administration was found.

Furthermore, it should be mentioned that — strictly speaking — the existence of highly anxious and high achieving testees contradicts the premise of the deficit-hypothesis itself [2, 3].

Table B5. Model fit and comparisons of nested models of interference effects in the interference-reduction approach.

Test sessions with modeled interference effects	$\Delta\chi^2(df)$	p	$\chi^2(df)$	p	χ^2/df	RMSEA [90%CI]	CFI	TLI
1 to 7	-	-	10570.021 (7753)	< .001	1.363	.036 [.035, .038]	.970	.969
1 to 6	15.162 (13)	.297	10839.979 (7766)	< .001	1.396	.038 [.036, .040]	.967	.966
1 to 5	10.170 (13)	.680	10998.222 (7779)	< .001	1.414	.039 [.037, .040]	.965	.965
1 to 4	10.646 (13)	.640	11166.034 (7792)	< .001	1.433	.040 [.038, .041]	.964	.963
1 to 3	15.050 (13)	.304	11421.533 (7805)	< .001	1.463	.041 [.039, .043]	.961	.961
1 and 2	15.098 (13)	.301	11690.061 (7818)	< .001	1.495	.042 [.041, .044]	.958	.958
1	18.883 (13)	.127	12031.721 (7831)	< .001	1.536	.044 [.043, .046]	.955	.955
None	33.614 (13)	.001	12701.681 (7844)	< .001	1.619	.047 [.046, .049]	.948	.948

Notes. *df* = degrees of freedom; RMSEA = Root Mean Square Error of Approximation; CI = Confidence Interval; CFI = Comparative Fit Index; TLI = Tucker-Lewis Index. Models were identified by setting the variances of latent variables to 1. Participants with ceiling effects were included in the analysis (N = 276).

Table B6. Two-sample Welch-tests of fear of failure sum scores between the main study sample and high achieving participants for every test session and cumulated across test administrations.

Test session	$Mean_{ms}$ (SD)	$Mean_{ha}$ (SD)	$t(df)$	one-tailed p-value
1	16.582 (6.200)	17.431 (5.828)	0.928 (77.797)	.178
2	15.116 (6.352)	16.627 (5.535)	1.712 (82.657)	.045
3	13.569 (6.001)	15.627 (5.810)	2.271 (76.112)	.013
4	12.929 (5.949)	14.392 (5.546)	1.678 (78.293)	.049
5	12.480 (6.150)	13.686 (5.202)	1.443 (84.801)	.076
6	12.360 (6.005)	13.549 (5.551)	1.360 (78.809)	.088
7	11.889 (6.014)	13.549 (5.143)	2.014 (83.996)	.024
grand	94.924 (38.714)	104.863 (35.092)	1.791 (80.033)	.039

Notes. ms = main study sample (N = 225); ha = high achievers (participants with ceiling effects, N = 51); SD = Standard Deviation; *df* = degrees of freedom; grand = cumulated fear of failure sum scores across test sessions.

Appendix C

An alternative strategy for model identification

Here we present the results for interference and deficit detection when models are identified in a way that follows the original strategy put forward by Halpin et al. [4]. Only configural longitudinal measurement invariance is imposed on the model, but all interference effects of a respective test session are restricted to be equal. This method of model identification leads to the most conservative approach for interference detection in the AT-model framework [4]. Note, however, that configural invariant repeated measurement of cognitive ability does not allow for the inclusion of latent difference-variables. Instead, interference-reduction was investigated via the LAT model (Figure 2). Although this model does not estimate retest effects, it still allows for an investigation of the reduction of interference across test administrations.

Table C1 displays comparisons of nested models in the interference-reduction approach for the main study sample (N = 225). The second column of the table shows the estimated value for the interference effects in the last test session with assumed interference of the respective row. The third column displays the deficit effect for that session. These values are taken from the model that includes interference effects at all test sessions. Table C2 delivers the same information for the sample including participants with ceiling effects (N = 276).

Conclusions regarding the presence of interference and the lack of observed deficits remain the same for the respective samples when implementing such conservative constraints on interference effects.

Table C1. Model fit and comparisons of nested models of interference effects in the interference-reduction approach with restricted interference effects (main study sample).

Test sessions with modeled interference effects	IE	DE	$\Delta\chi^2(df)$	*p*	$\chi^2(df)$	*p*	χ^2/df	RMSEA [90% CI]	CFI	TLI
1 to 7	-0.050	-.021	-	-	7510.542 (7658)	.884	0.981	.000 [.000, .006]	1.000	1.000
1 to 6	-0.045	-.032	0.810 (1)	.368	7553.653 (7659)	.802	0.986	.000 [.000, .008]	1.000	1.000
1 to 5	-0.023	-.014	0.554 (1)	.457	7589.598 (7660)	.714	0.991	.000 [.000, .009]	1.000	1.000
1 to 4	-0.077	.031	0.177 (1)	.674	7598.895 (7661)	.691	0.992	.000 [.000, .009]	1.000	1.000
1 to 3	-0.081	-.038	2.259 (1)	.133	7707.853 (7662)	.354	1.006	.000 [.000, .012]	.999	.999
1 and 2	-0.093*	.016	2.246 (1)	.134	7834.787 (7663)	.083	1.022	.010 [.000, .015]	.998	.997
1	-0.168**	.053	3.950 (1)	.047	7994.406 (7664)	.004	1.043	.014 [.008, .018]	.995	.995
None	-	-	7.352 (1)	.007	8435.005 (7665)	< .001	1.100	.021 [.018, .024]	.989	.989

Notes. $N = 225$. IE = Interference Effect; DE = Deficit Effect; *df* = degrees of freedom; RMSEA = Root Mean Square Error of Approximation; CI = Confidence Interval; CFI = Comparative Fit Index; TLI = Tucker-Lewis Index. Models were identified by setting the variances of latent variables to 1 and by restricting all interference effects of a respective test session to the same value.
$^*p < .05$; $^{**}p < .01$

Table C2. Model fit and comparisons of nested models of interference effects in the interference-reduction approach with restricted interference effects (complete sample).

Test sessions with modeled interference effects	IE	DE	$\Delta\chi^2(df)$	p	$\chi^2(df)$	p	χ^2/df	RMSEA [90%CI]	CFI	TLI
1 to 7	-0.009	-.011	-	-	7742.575 (7658)	.246	1.011	.006 [.000, .012]	.999	.999
1 to 6	-0.001	-.052	0.027 (1)	.870	7744.039 (7659)	.245	1.011	.006 [.000, .012]	.999	.999
1 to 5	0.014	-.016	< 0.001 (1)	.984	7744.066 (7660)	.248	1.011	.006 [.000, .012]	.999	.999
1 to 4	-0.041	.034	0.085 (1)	.771	7748.617 (7661)	.239	1.011	.006 [.000, .012]	.999	.999
1 to 3	-0.044	.003	0.702 (1)	.402	7784.642 (7662)	.161	1.016	.008 [.000, .013]	.999	.999
1 and 2	-0.060	.007	0.760 (1)	.383	7829.088 (7663)	.091	1.022	.009 [.000, .013]	.998	.998
1	-0.121*	.014	1.878 (1)	.171	7911.916 (7664)	.023	1.032	.011 [.004, .015]	.997	.997
None	-	-	4.769 (1)	.029	8206.622 (7665)	< .001	1.071	.016 [.012, .019]	.994	.994

Notes. N = 276. IE = Interference Effect; DE = Deficit Effect; df = degrees of freedom; RMSEA = Root Mean Square Error of Approximation; CI = Confidence Interval; CFI = Comparative Fit Index; TLI = Tucker-Lewis Index. Models were identified by setting the variances of latent variables to 1 and by restricting all interference effects of a respective test session to the same value.
*p < .05

Appendix D

Retest effects as estimated with a simple neighbor-change model

Lastly, we present results for the retest effects when estimated within a simple neighbor-change model without latent anxiety variables. These shall demonstrate that retest effect estimates are not substantially affected by the modeling of interference effects. Figure D1 displays the retest effects from the simple neighbor-change model when estimated with the main study sample (N = 225). Figure D2 depicts the same results for the complete sample (N = 276). The numerical values indeed differ only slightly from the ones presented in Figure 6 and Figure B1. Hypothesis-decisions remain completely unaffected.

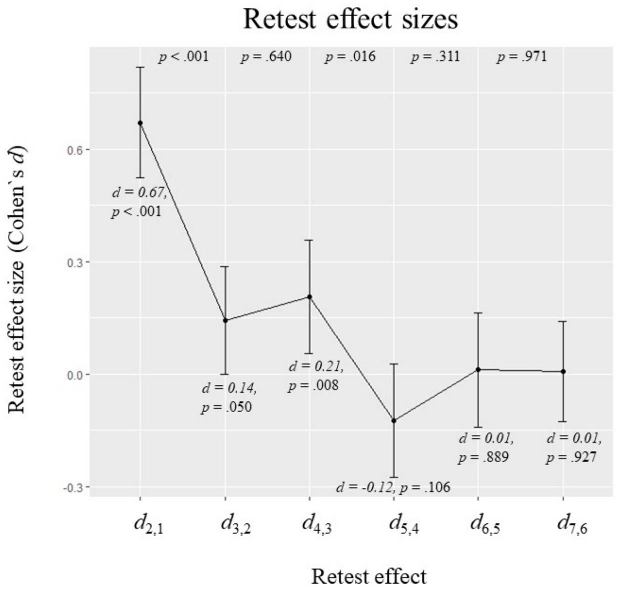

Figure D1. Means of the standardized latent difference variables of a simple neighbor-change model estimated within the main study sample (N = 225). They can be interpreted as retest effect sizes in terms on Cohen`s d between two successive test administrations. To obtain these parameters the model was identified by setting the variances of the latent variables to 1. $d_{2,1}$ represents the retest effect from the first to the second test administration. The remaining d are to be understood accordingly. Error-bars indicate two-tailed 95%-confidence intervals. p-values at the top relate to the difference between the respective successive retest effects.

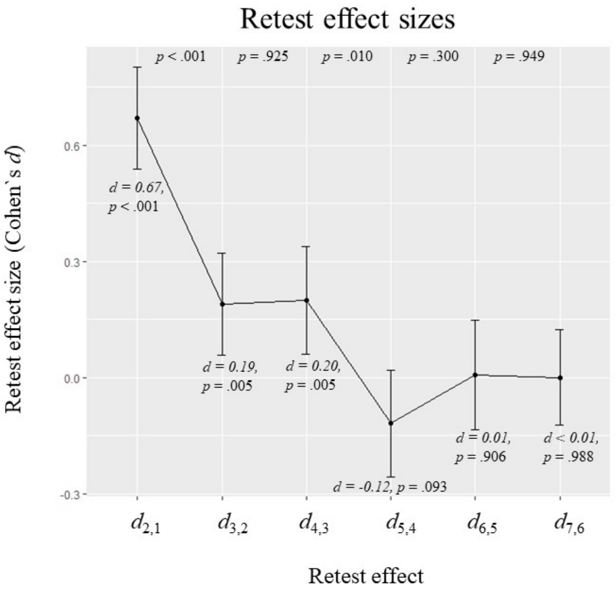

Figure D2. Means of the standardized latent difference variables of a simple neighbor-change model estimated within the complete sample (*N* = 276). They can be interpreted as retest effect sizes in terms on Cohen`s *d* between two successive test administrations. To obtain these parameters the model was identified by setting the variances of the latent variables to 1. $d_{2,1}$ represents the retest effect from the first to the second test administration. The remaining *d* are to be understood accordingly. Error-bars indicate two-tailed 95%-confidence intervals. *p*-values at the top relate to the difference between the respective successive retest effects.

References

1. Freund, P. A.; Hofer, S.; Holling, H. Explaining and Controlling for the Psychometric Properties of Computer-Generated Figural Matrix Items. *Applied Psychological Measurement* **2008**, 32, 195–210. DOI: 10.1177/0146621607306972
2. Oostdam, R.; Meijer, J. Influence of Test Anxiety on Measurement of Intelligence. *Psychological Reports* **2003**, 92, 3–20. DOI: 10.2466/pr0.2003.92.1.3
3. Tobias, S. Test Anxiety: Interference, Defective Skills, and Cognitive Capacity. *Educational Psychologist* **1985**, 20, 135–142. DOI: 10.1207/s15326985ep2003_3
4. Halpin, P. F.; da-Silva, C.; De Boeck, P. A. Confirmatory Factor Analysis Approach to Test Anxiety. *Structural Equation Modeling: A Multidisciplinary Journal* **2014**, 21, 455–467. DOI: 10.1080/10705511.2014.915377
